# Efficient Improvement of Eugenol Water Solubility by Spray Drying Encapsulation in Soluplus^®^ and Lutrol F 127

**DOI:** 10.3390/ph17091156

**Published:** 2024-08-31

**Authors:** Iskra Z. Koleva, Christo T. Tzachev

**Affiliations:** Faculty of Chemistry and Pharmacy, Sofia University “St. Kliment Ohridski”, 1 J. Bourchier Blvd., 1164 Sofia, Bulgaria; ohtct@chem.uni-sofia.bg

**Keywords:** eugenol, Soluplus^®^, Lutrol F 127, *myo*-inositol, Aerosil^®^ 200, low-temperature spray drying, encapsulation, green technology

## Abstract

Herein, we present an elegant and simple method for significant improvement of eugenol water solubility using the polymers Soluplus^®^ and Lutrol F 127 as carriers and spray drying as an encapsulation method. The formulations were optimized by adding *myo*-inositol—a sweetening agent—and Aerosil^®^ 200 (colloidal, fumed silica)—an anticaking agent. The highest encapsulation efficiency of 97.9–98.2% was found for the samples containing 5% eugenol with respect to the mass of Soluplus^®^. The encapsulation efficiencies of the spray-dried samples with 15% eugenol are around 90%. Although lowering the yield, the addition of Lutrol F 127 results in a more regular particle shape and enhanced powder flowability. The presence of Aerosil^®^ 200 and *myo*-inositol also improves the rheological powder properties. The obtained formulations can be used in various dosage forms like powders, granules, capsules, creams, and gels.

## 1. Introduction

Eugenol is a natural liquid compound with a spicy flavor and pleasant strongly aromatic scent. Its name originates from the old name of clove *Eugenia caryophyllata (Syzygium aromaticum* is the current one), since eugenol represents around 89% of the clove oil [1]. Other aromatic plants such as cinnamon, nutmeg, and basil also contain eugenol. Eugenol not only finds application in the food industry, cosmetics, and as a pesticide in agriculture, but also due to its antioxidant, antifungal, antibacterial properties, and even analgetic effect [2,3,4,5,6,7,8,9,10,11,12,13] is beneficial for pharmaceutical use. Eugenol and its derivates show in vitro activity against *Mycobacterium tuberculosis* and non-tuberculous mycobacteria [14]. For more than 90 years, in combination with ZnO, it has been used in dental medicine as a root canal sealer (endodontic sealer) [15,16]. However, eugenol is practically insoluble in water (less than 1 mg/mL at 20 °C) [17], which limits its bioavailability. An elegant way to improve its water solubility is by encapsulation in a water-soluble substance(s). Polymers such as Soluplus^®^ and Lutrol F 127 are very suitable candidates for this purpose, since they are water-soluble and are approved for pharmaceutical use. Furthermore, Soluplus^®^ is developed by BASF with the purpose to act as a matrix polymer for solid solutions and due to its amphiphilic chemical nature is capable of solubilizing poorly soluble drugs in aqueous media [18,19,20,21,22,23]. Lutrol F 127, also created by BASF, is a kind of poloxamer—a copolymer of ethylene and propylene oxides. Since it has an influence on the viscosity, it can be applied as a stabilizer for topically administrated suspensions and finds application in toothpastes and mouthwashes [24]. Its excellent solubilizing properties are useful for active substances with low solubility and it could also be applied to essential oils [24].

Spray drying is a widely used technique, which allows dry powders to be obtained from liquid solutions in one step [25,26,27,28]. It is used in the chemical, food, and pharmaceutical industries [29]. The speed of the process and the short drying time allow drying without degradation of even temperature-sensitive substances [30,31]. Several studies report the encapsulation of drugs and substances with therapeutic potential in Soluplus^®^ by spray drying [18,32,33,34], but so far there is a lack of data in the literature on loading eugenol in Soluplus^®^ and Lutrol F 127 using spray drying or other methods.

Peng et al. improved eugenol solubility by nanoliposome encapsulation using ethanol injection and dynamic high-pressure microfluidization [35]. The encapsulation efficiency was 59.2 ± 4.7%. Enhancement of the solubility of eugenol was also achieved by β-cyclodextrin inclusion complexes [36,37,38]. Talón and co-authors encapsulated eugenol by spray drying using soy lecithin and whey protein for the purpose of preparing antioxidant and antimicrobial powders for food applications [39]. They also studied how the addition of maltodextrin, oleic acid, and chitosan would influence the encapsulation efficiency, thermal stability, and release kinetics, as well as the antioxidant and antimicrobial activities of eugenol. Using UV-VIS spectroscopy they determined the encapsulation efficiency and found higher encapsulation efficiencies of 95–98% for the formulations with only soy lecithin or whey protein. Bittencourt and co-authors also loaded eugenol by spray drying in low-lactose whey protein, bovine serum, rice bran protein, albumin, and carrageenan [40]. The best encapsulation of 80.5% was established for the formulation with bran protein, bovine serum albumin, and carrageenan. Encapsulation of clove oil in casein by the spray drying method with an encapsulation efficiency of 97.78% was also reported [41]. Woranuch and co-workers improved the thermal stability of eugenol by encapsulation into chitosan nanoparticles using an emulsion–ionic gelation cross-linking method with a highest encapsulation efficiency of 20% [42]. Paulo and Santos loaded eugenol in an ethyl cellulose polymer-based matrix by a double emulsion solvent evaporation technique and achieved an encapsulation efficiency of 94.7% [43]. Food polymers—zein, sodium caseinate, and pectin—were also used to encapsulate eugenol and stable nanoparticles were obtained via a heat- and pH-induced complexation process [44]. Shao et al. obtained eugenol-loaded nanoemulsions using the ionotropic gelation method with ultrasound-mediated emulsification and chitosan as a carrier with an encapsulation efficiency of up to 12% [45].

*Myo*-inositol is an isomer of cyclohexanehexol, which is biosynthesized from glucose in our body [46]. Impressive benefits from the intake of inositol have been reported like relieving symptoms of polycystic ovary syndrome, lowering the serum insulin level, and improving insulin resistance [47]. In spray drying powder technology, as a member of the group of sugar alcohols, it acts as a sweetening, hydrophilizing, and powder structure-forming agent. Aerosil, a colloidal silicon dioxide, is widely used as an excipient in pharmaceutical formulations, food products, and cosmetics [48]. In spray drying powder technology, colloidal silicon dioxide acts as a dispersion stabilizer, pore-forming, and powder gliding agent.

This study aims to increase the water solubility of eugenol by encapsulation in Soluplus^®^ and Lutrol F 127 using green, high-yield low-temperature spray drying technology using pharmacopoeial and GRAS (generally recognized as safe) listed active substances and excipients. The solvent selected for the spray drying solutions was water, which is the greenest possible solvent. Formulations containing *myo*-inositol and Aerosil^®^ 200 were prepared to study how the quantity of the entrapped eugenol, the morphology, and the size of the spray-dried particles will be influenced. Additionally, the effect of the spray drying inlet/outlet temperatures was also considered for selected compositions using the following combinations 90/45 °C, 80/41 °C, and 70/30 °C. The amount of the embedded eugenol in the obtained spray-dried samples was estimated using UV/VIS spectroscopy and the dissolution profiles of selected spray-dried powders were compared. The morphology was studied via scanning electron microscopy (SEM) and the size of the particles in a water solution was determined by dynamic light scattering (DLS). The samples were also characterized using Fourier-transform infrared spectroscopy (FTIR) and differential scanning calorimetry (DSC).

## 2. Results and Discussion

### 2.1. Solubilization Capacity and Stability of the Emulsions for Spray Drying

**Figure 1:**
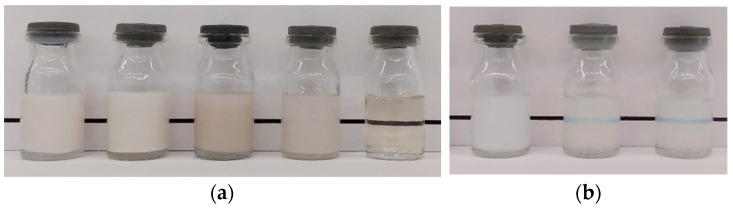
Water solutions of (**a**) eugenol (E) and Soluplus^®^ (S)/Lutrol F 127 (L). The ratios from left to right are the following: E:S = 1:1; E:S = 1:2; E:L = 1:1; E:L = 1:1.33; and E:L = 1:2. Only in the case of E:L = 1:2 is no precipitate observed; (**b**) selected spray-dried solid powders (200 mg in 5 mL H_2_O) with different amount of eugenol with respect to the mass of Soluplus^®^: from left to right, 15% eugenol, 10% eugenol and 5% eugenol—no precipitate is formed. The solution’s turbidity is due to the high Soluplus^®^ concentration and the increased size of the Soluplus^®^ micelles due to the presence of eugenol.

First, we studied the solubilization capacity of Soluplus^®^ (S) and Lutrol F 127 (L) separately towards eugenol (E) by variation of the amount of eugenol added to water solutions of the polymers. The following ratios between the masses of eugenol and the polymers were tested: E:S = 1:1; E:S = 1:2; E:S = 1:4; E:S = 1:5; E:S = 1:6.(6); E:L = 1:1; E:L = 1:1.33; and E:L = 1:2 (Figure 1a). No precipitation is observed only when the mass of eugenol is 15% with respect to the weight of the Soluplus^®^—E:S = 1:6.(6). In the case of Lutrol F 127, there is no precipitate even at a ratio of E:L = 1:2 and the solution is transparent indicating that the solubilizing properties of Lutrol F 127 are superior compared to those of Soluplus^®^. Based on the obtained results for the stability of the emulsions, for the spray drying feeding solutions, which contain Lutrol F 127, the ratio E:L = 1:2 was selected. For the formulations that contain only Soluplus^®^, the ratios selected were E:S = 1:6.(6), E:S = 1:10, and E:S = 1:20 corresponding to 15%, 10%, and 5% eugenol to the weight of Soluplus^®^ (see Section 3.2).

### 2.2. Spray-Dried Powders

#### 2.2.1. Morphology of the Particles

**Figure 2:**
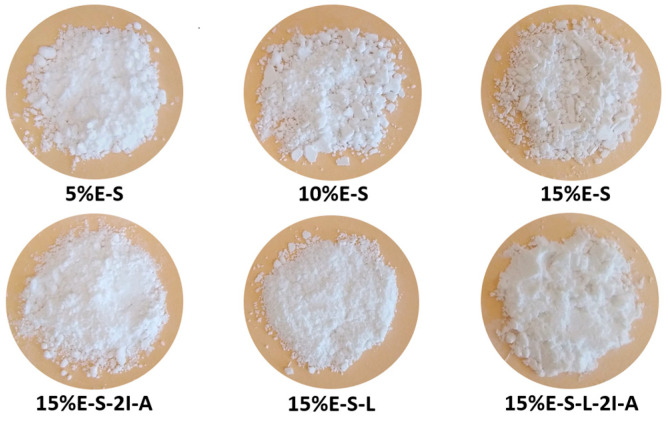
Spray-dried powders of selected formulations. The rest of the compositions are shown in Supplementary Materials, Figure S1.

Selected samples are shown in Figure 2 and the rest of the powders in Figure S1. The spray-dried powders are white colored and uniform in appearance. However, some particle aggregation is observed, due to their hygroscopic properties, which is much less pronounced, when Lutrol F 127 is included in the formulation. Lutrol F 127 was used only in combination with Soluplus^®^, since due to its low melting temperature, it stuck on the drying chamber and the cyclone separator and it was not possible to obtain powders. The powders containing *myo*-inositol and Aerosil^®^ 200 are also less prone to aggregate formation. The loss on drying of the different formulations is in the range of 2–4% (Table S1).

The water solutions of the formulations are clear with the characteristic opalescence of Soluplus^®^ micelles as no precipitation is observed (Figure 1b). However, the water solution of the 15%E-S formulation looks turbid, because the higher concentration of eugenol in the Soluplus^®^ micelles increases their size. In water media, it is expected that the hydrophobic vinyl acetate and the vinyl caprolactam fragments will be located in the core of the micelles surrounding the eugenol molecules, while the hydrophilic PEG chains will be oriented towards the water molecules. The appearance of the water solutions of the spray-dried formulations containing *myo*-inositol and/or Aerosil^®^ 200 is very similar to that of the 15%E-S.

**Figure 3:**
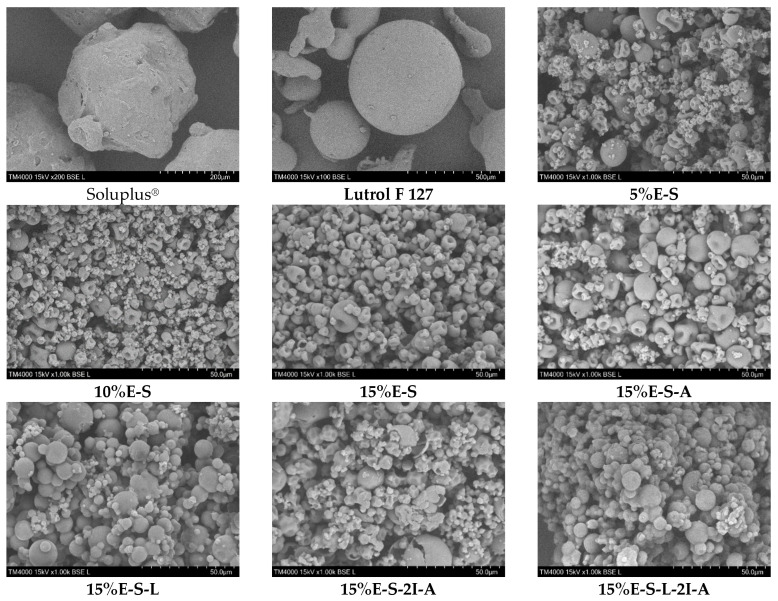
SEM micrographs of Soluplus^®^ and Lutrol F 127 before spray drying and selected samples obtained at 70/30 °C inlet/outlet temperatures.

The morphology of the spray-dried powders was studied via scanning electron microscopy (SEM). The SEM images of selected samples show that after spray drying the size of the particles is dramatically reduced as particles with diverse shapes and sizes are observed (Figure 3). For example, the particles in the raw Soluplus^®^ and Lutrol 127 are in the order of 300 μm and 500 μm, respectively, while in the spray-dried formulations, the size of the bigger particles is roughly around 10 μm as the smallest particles are approximately 2 μm (Supplementary Materials, Figure S2). In some cases, single particles even below 1 μm are observed (Supplementary Materials, Figure S2).

**Figure 4:**
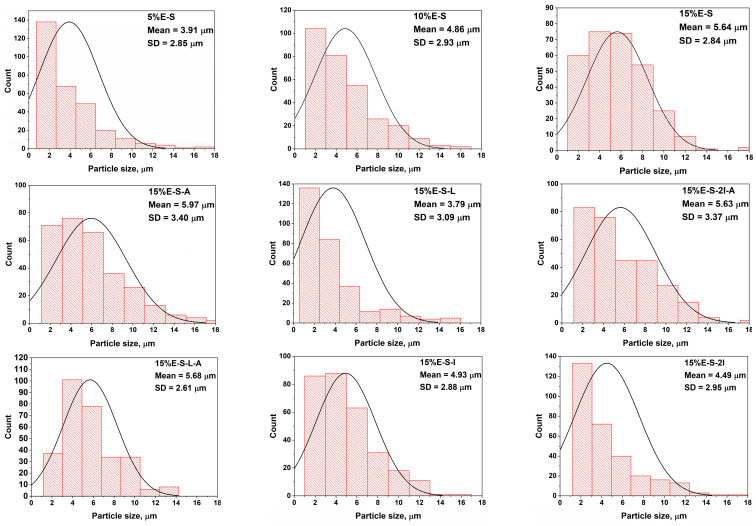
Particle size distribution of selected spray-dried formulations. The average value of the diameter of the particles and the standard deviation (SD) are also shown.

The average particle size varies in the range of 3.79—5.97 µm (Figure 4). Some deformation of the particles is observed due to the evaporation of water during the spray drying process. It seems that the addition of *myo*-inositol and Aerosil^®^ does not significantly change the morphology. At the same time, the presence of Lutrol F 127 leads to a more well-defined and spherical shape of the particles. However, it should be noted that in the case of Aerosil^®^ 200-containing powders, less particle deformation and a greater number of particles with a regular shape are observed. The inlet/outlet spray drying temperatures do not considerably influence the shape of the particles (see Supplementary Materials, Figure S2—SEM images of samples 15%E-S obtained at 70, 80, and 90 °C inlet temperatures).

The obtained ^1^H NMR spectrum of the 15%E-S formulation suggests that no degradation products are formed during the spray drying process as no additional signals are observed but only the typical one of eugenol and Soluplus^®^ protons (Figure S3). The ^1^H NMR Soluplus^®^ spectrum is fully in line with that obtained in previous research [49].

#### 2.2.2. Encapsulation Efficiency

**Figure 5:**
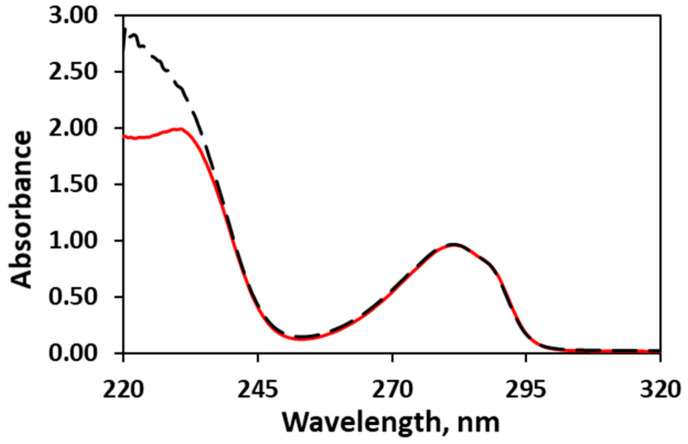
UV spectrum of eugenol in ethanol (red line—pristine form, black dotted line—eugenol encapsulated in Soluplus^®^, sample 15%E-S).

**Figure 6:**
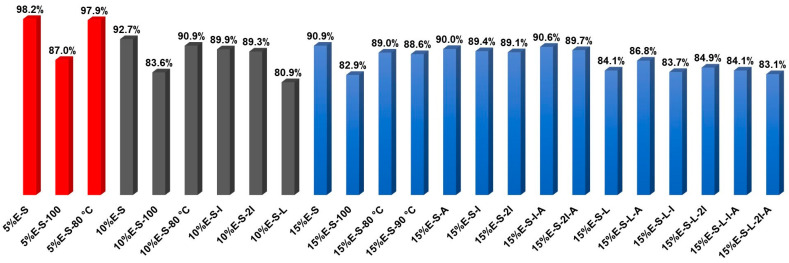
Graphical representation of the encapsulation efficiency of the formulations shown in Table 1. For visual clarity, the compositions with different amounts of eugenol are expressed by colors: red—5%, black—10%, and blue—15% eugenol.

The quantity of the encapsulated eugenol in the spray-dried samples was estimated using UV-VIS spectroscopy. Eugenol absorbs in the UV region as the longest wavelength maximum is observed at 282 nm (Figure 5). The shape as well as the maximum of the band are preserved in the UV spectra of the spray-dried samples (Figure 5). The results obtained for the amount of entrapped eugenol in the polymer matrix are presented in Table 1 and Figure 6. In general, the amount of loaded eugenol in the solutions for spray drying as well as the ratio between the solids and water are crucial for the encapsulation efficiency. The encapsulation efficiency is also strongly influenced by the presence of Lutrol F 127 in the composition. The encapsulation efficiency is highest, 97.9–98.2%, in the case of the samples that contain 5% eugenol (ca. 0.2 g) with respect to the mass of Soluplus^®^. However, the encapsulation efficiency of the 10%E-S and 15%E-S formulations is also satisfactory—in the range of 89.3–92.7% for the samples obtained from solutions with a ratio of solids to water of 1:10. The lower percentage of loaded eugenol compared to the composition with 5% eugenol might be due to an amount of eugenol, which was outside the micelles of Soluplus^®^ and during the spray drying process was released in the drying chamber. To clarify this issue, the concentration of eugenol (expressed as a percentage of the added eugenol) in the solutions for spray drying for the 5%-S, 10%-S, and 15%-S formulations was measured using UV/VIS spectroscopy and the following results were obtained: 99%, 96%, and 93%. Although no dense precipitate is observed in the spray drying feed solutions, upon close inspection in strong light in the case of 10% and 15% eugenol, very small non-dissolved pieces of eugenol are visible.

In the case of the samples obtained from more diluted solutions—solids:water = 1:25—the encapsulations efficiency is lowered by around 11%, 9%, and 8% for 5%E-S, 10%E-S, and 15%E-S formulations, respectively (Table 1, Figure 6). The variations of the spray drying inlet/outlet temperatures do not significantly affect the entrapment efficiency. For example, in the case of 15%E-S samples obtained at 70/30, 80/41, and 90/45 °C inlet/outlet temperatures, the corresponding encapsulation efficiencies are 90.9, 89.0, and 88.6% and the differences are comparable to the values of standard deviations, which are around 2%. Although the encapsulation efficiency of the formulations with 15% eugenol is lower—around 90%—the overall amount of the loaded eugenol is larger compared to formulations containing loaded eugenol in different carriers obtained in other studies. For example, the eugenol loading was 0.065 g/g total solids using soy lecithin and whey protein [39], while in the case of the obtained formulations in the current study is around 0.130 g/g total polymers.

In the case of the powders that contain Lutrol F 127 the efficiency is lower, ~84%, most likely because during the spray drying process, due to the low melting temperature of Lutrol F 127 (Tm ~ 56 °C), part of the eugenol is released and stuck in the drying chamber as well as the cyclone separator. The same applies to the sample recovery, which is lowered to 30–40%, while for the samples without Lutrol F 127, the yield is ~50–60% (see Section 3.3). The addition of *myo*-inositol does not change the encapsulation efficiency, since for the formulations without Lutrol F 127 it is in the range of 89.1–90.6%.

The encapsulation efficiencies of 15%E-S-I-A and 15%E-S-2I-A formulations are not the highest but are very close to that of the 15%E-S composition and, since they possess better rheological properties, could be considered as optimal among all studied formulations.

#### 2.2.3. FTIR Analysis

**Figure 7:**
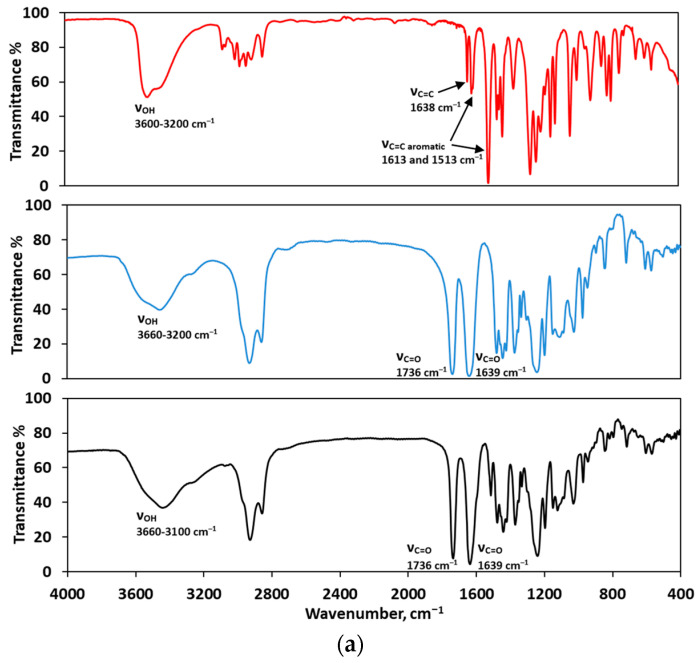

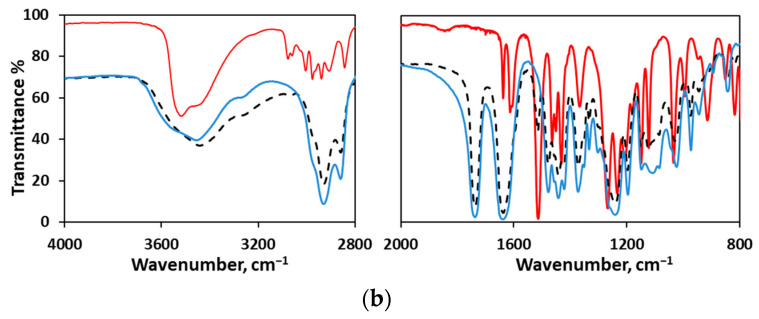
FTIR spectra of (**a**) eugenol (red line, liquid film), Soluplus^®^ (blue line, KBr disk), and eugenol encapsulated in Soluplus^®^, sample 15%E-S (black dotted line, KBr disk) and (**b**) comparison of the FTIR spectra in selected ranges.

The FTIR spectrum of a selected spray-dried sample containing eugenol and Soluplus^®^, 15%E-S, confirms the presence of eugenol (Figure 7, black line). In the IR spectrum of the pure eugenol in the region 3600–3200 cm^–1^, a wide band is observed, which corresponds to O–H stretching vibration as the widening is due to the presence of hydrogen bonds (Figure 7, red line). The aromatic C–H stretching vibrations appear at 3075 and 3058 cm^–1^, while the methyl and methylene C–H stretching vibrations are in the region 3000–2800 cm^–1^. The band at 1513 cm^–1^ is assigned to the ring stretching vibrations, ν(C=C). Such a band is absent in the spectrum of the pure Soluplus^®^ but present in the spectrum of the spray-dried sample, 15%E-S. The stretching vibrations of the ester and the amide carbonyl groups are exhibited at 1736 cm^–1^ and 1639 cm^–1^, respectively. The stretching vibrations of the OH groups in the IR spectrum of Soluplus^®^ appear in the region 3660–3200 cm^–1^, while in the spectrum of the sample 15%E-S is observed a slight shift to lower frequencies, 3100 cm^–1^, due to formation of hydrogen bonds between Soluplus^®^ and eugenol. Since there is no shift of the C=O stretching bands in the IR spectrum of the sample, most likely in the solid state hydrogen bonds between the eugenol OH groups and the Soluplus^®^ OH groups are formed.

#### 2.2.4. DSC Analysis

**Figure 8:**
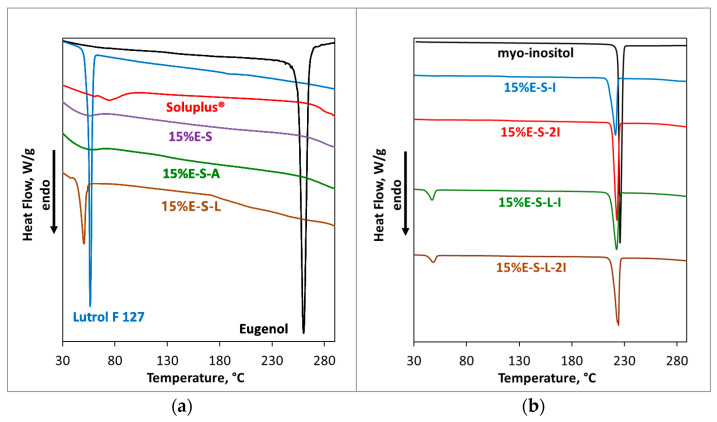
DSC thermograms of (**a**) eugenol, Soluplus^®^, Lutrol F 127, and selected powders; (**b**) *myo*-inositol and selected powders containing *myo*-inositol.

The DSC thermogram of pristine eugenol shows a sharp endothermic peak at 255.6 °C (Figure 8), which corresponds to its boiling point and is consistent with that obtained by Paulo et al. [43], 254.0 °C. In the thermogram of pure Soluplus^®^, the broad peak at 64.9–75.2 °C corresponds to its glass transition (Tg) and is in agreement with the stated value of ~70 °C in the technical information provided by BASF [18]. In the DSC of Lutrol F 127, a sharp peak at 56.3 °C is observed, which is also in line with the provided Tm from BASF [24]. The absence of the sharp characteristic endothermic peak of pure eugenol in the DSC thermograms of the spray-dried samples suggests that the eugenol is successfully embedded in the polymer matrix (Figure 8, samples 15%E-S, 15%E-S-A, and 15%E-S-L). In the DSC of 15%E-S-L, the peak of Lutrol F 127 is shifted to 50.3 °C due to the presence of eugenol and Soluplus^®^. The thermograms of the samples with lower eugenol content—5% and 10% (Supplementary Materials, Figure S4)—and with Aerosil^®^ 200—15%E-S-A— are similar to that of 15%E-S. For pure *myo*-inositol, a characteristic sharp peak is observed at 225.8 °C, which corresponds to its melting point (Figure 8). The peak is slightly shifted to around 222 °C in the DSC thermograms of the formulations with inositol due to the presence of the polymers and eugenol as the latter is loaded in the polymer matrix due to the absence of a peak at 255.6 °C. In the thermograms of 15%E-S-L-I and 15%E-S-L-2I, samples peaks at 47.3 and 48.3 °C are visible, respectively, corresponding to the Tg of Lutrol F 127.

#### 2.2.5. Particle Size in a Water Solution

The size of the particles of the spray-dried samples in a water solution was measured using DLS (Table 2). The average size of the particles of the formulations with different percentages of eugenol—5%, 10%, and 15%—and Soluplus^®^ are 66.4 ± 2.1, 68.7 ± 2.2, and 64.5 ± 1.8 nm, respectively. A slight increase in size to 69.2–82.8 nm is observed in the cases in which Lutrol F 127, *myo*-inositol, and/or Aerosil^®^ 200 are present in the composition. However, in the case of 15%E-S-A and 15%E-S-2I formulations, the particles are slightly smaller, 63.1 and 66.1 nm, respectively. The averaged polydispersity index (PDI) describes the width of particle size distribution and for all samples is in the interval 0.075–0.299 and below 0.7 (even in cases in which the standard deviation is in the order of the averaged value) indicating a uniform and narrow distribution of the particle size. The PDI of the samples containing only Soluplus^®^ is around 0.080 and is approximately two times lower compared to the formulations with Lutrol F 127, *myo*-inositol, and Aerosil^®^ 200.

One can notice that there is a significant difference in the particle sizes in the solid state (see Figure 3, SEM micrographs) and in a water solution. The estimated hydrodynamic diameter corresponds to the size of the formed Soluplus^®^ micelles in an aqueous solution, while the powder particles are formed by the evaporation of the water droplets from the micelle solution resulting in a larger size.

#### 2.2.6. Dissolving Properties of the Spray-Dried Powders

**Figure 9:**
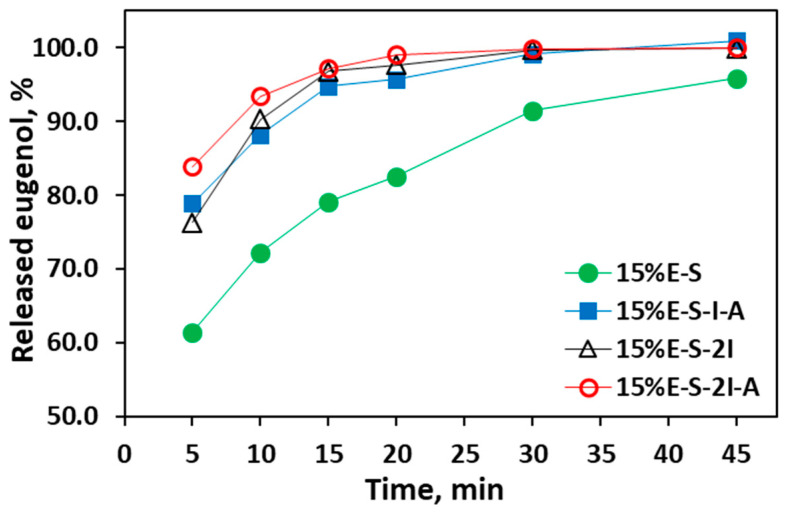
Dissolution profiles of eugenol from selected spray-dried formulations in water media at 25 °C and 50 rpm.

To evaluate the powder’s immediate dissolving properties, dissolution profiles of the 15%E-S, 15%E-S-I-A, 15%E-S-2I, and 15%E-S-2I-A formulations in water at room temperature were obtained (Figure 9). It is expected that the eugenol release is determined by the dissolution speed of Soluplus^®^, since in a water solution, the eugenol is located in the Soluplus^®^ micelles. At the 5th minute above 60% of the eugenol from all studied powders is dissolved and at the 10th minute above 85% for the formulations containing *myo*-inositol. The released eugenol at the 15th minute is 79.1%, 94.8, 96.7%, and 97.1% for the 15%E-S, 15%E-S-I-A,15%E-S-2I, and 15%E-S-2I-A formulations, respectively. This demonstrates that *myo*-inositol not only improves the powders’ flowability and reduces their agglomeration, but also enhances eugenol’s dissolution rate, thus obtaining rapidly soluble eugenol.

## 3. Materials and Methods

### 3.1. Materials and Reagents

**Figure 10:**
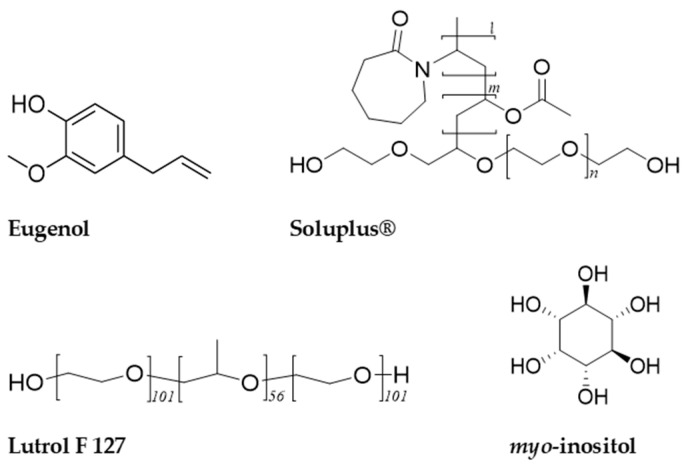
Chemical structures of eugenol, Soluplus^®^, Lutrol F 127, and *myo*-inositol.

Eugenol (AlfaAesar, 99 % purity), Soluplus^®^, and Lutrol F 127 (BASF SE, Ludwigshafen, Germany) were used (Figure 10). Ethanol Absolute 99.8+%, Certified AR for Analysis (Fisher Chemical™), *myo*-inositol, and Aerosil 200 (colloidal silica) were also used.

### 3.2. Preparation of the Solutions for Spray Drying (Summarized in Supplementary Materials, Table S2, and in Table S3 the Excipients Are Expressed as Fractions Considering That the Sum of All Excipients Is 1.000 g)

#### 3.2.1. Formulations Only with Soluplus^®^—5%E-S, 10%E-S, and 15%E-S

Soluplus^®^ was dissolved in distilled water (4.000 g per 40.0 mL H_2_O) using a magnetic stirrer at 400 rpm for 10 min until dissolved completely. Then, the polymer solution was added to the eugenol and stirred at 400 rpm for 3 h at room temperature for the formulations with 5% eugenol (0.20 g) and 6 h for the formulations with 10% (0.40 g) and 15% (0.60 g) eugenol with respect to the mass of the Soluplus^®^.

#### 3.2.2. Formulations Containing Lutrol F 127 and 10%/15% Eugenol with Respect to the Total Mass of the Polymers—10%E-S-L and 15%E-S-L and 15%E-S-L-I, 15%E-S-L-2I, 15%E-S-L-I-A, and 15%E-S-L-2I-A

Lutrol F 127 was dissolved in distilled water (1.200 g for 15%E and 0.800 g for 10%E per 20.0 mL H_2_O) using a magnetic stirrer at 400 rpm for 10 min until dissolved completely. Then, the polymer solution was added to the eugenol (0.60 g eugenol for 15%E and 0.40 g for 10%E) and the solution was stirred at 400 rpm for 3 h at room temperature. Soluplus^®^ was dissolved in water (2.800 g and 3.600 g for 15%E and 10%E, respectively, per 20.0 mL H_2_O) using a magnetic stirrer at 400 rpm for 10 min and the obtained solution was mixed with the solution of eugenol and Lutrol F 127 and stirring continued for 1 h.

#### 3.2.3. Formulations containing *myo*-inositol and/or Aerosil^®^ 200—15%E-S-A, 15%E-S-L-A, 15%E-S-I, 15%E-S-2I, 15%E-S-L-I, 15%E-S-L-2I, 15%E-S-I-A, 15%E-S-2I-A, 15%E-S-L-I-A, and 15%E-S-L-2I-A:

Two different ratios of polymer(s) to *myo*-inositol 1:1 and 1:2 were used as to the solution of the polymer(s) and eugenol was added 4.000 g (denoted as I) or 8.000 g (denoted as 2I) *myo*-inositol, respectively, followed by dilution to 80.0 mL H_2_O and stirring continued for 1 h.

In the case of the formulations containing Aerosil^®^ 200, to the solution of the polymer(s) and eugenol was added 0.100 g Aerosil^®^ 200, followed by dilution to 80.0 mL H_2_O and stirring continued for 1 h.

### 3.3. Spray Drying Conditions

The spray-dried powders were obtained using a Büchi Mini spray dryer B-290 (Büchi Laboretechnik AG, Flawil, Switzerland). A nozzle with a diameter of 0.7 mm was used. The sample flow rate was 6 mL/min (pump rate 20%). The inlet temperature was 70 °C for most of the formulations and the outlet temperature was 41 ± 2 °C and inlet/outlet combinations of 90/45 °C and 80/41 °C were tested for selected formulations (see Table 1). The aspiration flow was 35 m^3^/h (aspiration 100%). The yield, calculated as the percentage of the collected mass of the spray-dried powders divided by the overall mass of the added compounds in the spray drying feeding solutions, was ~50% for the formulation only with Soluplus^®^, ~60% for the formulations containing *myo*-inositol and ~30–40% for the formulations with Lutrol F 127. The spray-dried powders were stored in darkness in plastic containers at room temperature.

### 3.4. Scanning Electron Microscopy (SEM) and Particle Size

The SEM images of the samples were obtained using a Hitachi TM4000 scanning electron microscope (Hitachi, Tokyo, Japan) with a BSE detector under a low vacuum. The samples were gold-coated before recording the SEM micrographs.

The particle size was determined using the ImageJ program as for each formulation the diameter of 300 particles from SEM images of different parts of the samples were measured.

### 3.5. Assay of the Encapsulated Eugenol (Encapsulation Efficiency and Entrapment Efficiency)

Approximately 100 mg of the spray-dried samples were dissolved in 25.0 mL ethanol. In the case of the samples that contain only Soluplus^®^ and Lutrol F 127, 0.5 mL of the solution was diluted to 10.0 mL with ethanol. The absorbance of Soluplus^®^ at a concentration close to corresponding to the solutions of the samples containing only Soluplus^®^ and Lutrol F 127 at 282 nm is 0.000 (Supplementary Materials, Figure S5). For the other samples containing *myo*-inositol and Aerosil^®^ 200, the stock solution was filtered with a 0.45 μm syringe filter and then 1.0 mL of the filtrate was diluted to 10.0 mL with ethanol. The absorbance was measured at the maximum 282 nm at room temperature using a UV-VIS spectrophotometer, Shimadzu UV-1800. Three assays were performed for each sample and the average value and standard deviation were calculated (Table 1).

The encapsulation efficiency (%) was calculated in the following way:

$$encapsulation\;efficiency\;(\%)=\frac{Encapsulated\;eugenol}{Theoretical\;eugenol}\times 100$$

where encapsulated eugenol is the amount of eugenol content determined by the absorption of the spray-dried sample in the UV spectrum and theoretical eugenol is the added amount of eugenol in the solutions for spray drying.

The stock solution for the calibration curve was prepared with 103.88 mg eugenol diluted with ethanol to 100.0 mL in a volumetric flask. Seven solutions were obtained from the stock solution by dilution to 25.0 mL of the following aliquots: 0.1, 0.3, 0.6, 0.9, 1.2, 1.5, and 2.0 mL. The absorbance of the solutions was measured at the maximum—282 nm (Supplementary Materials, Figure S6).

### 3.6. Fourier-Transform Infrared (FTIR) Spectroscopy

The FTIR spectra of the samples were recorded in the range 4000–400 cm^−1^ in transmittance mode using 80 scans and 4 cm^−1^ resolution using a Shimadzu FTIR-8400S spectrophotometer.

### 3.7. Differential Scanning Calorimetry (DSC)

DSC analysis was performed using a Q200 differential scanning calorimeter, TA Instruments, USA, over an interval of 25–350 °C at a rate of 5 °C/min under a nitrogen atmosphere with a flow rate of 50 mL/min. The samples were sealed in Tzero Aluminium pans and a Tzero Hermetic pan was used for pure eugenol.

### 3.8. Particle Size in Water Solution

The particle size and the polydispersity index (PDI) of the spray-dried formulations in distilled water solution (1 mg/mL) were measured by dynamic light scattering using a DLS Malvern 4700 c with a green laser at a wavelength of 532 nm. Each solution was measured ten times, and the obtained particle size and PDI values were averaged.

### 3.9. Loss on Drying (Water Content)

The water content was determined using a Biobase BM-50-5 Rapid Moisture Meter by heating 0.5 g powder at 105 °C to a constant mass. For the 15%-S-A composition, a test with different heating temperatures—60 °C, 80 °C, 105 °C, and 120 °C—was performed and the following results were obtained: 1.00%, 2.95%, 4.00%, and 4.00%, respectively. Based on this, a heating temperature of 105 °C was selected.

### 3.10. Dissolution of Eugenol from the Spray-Dried Powders

The release profiles were determined using an Erweka Dissolution Tester (Apparatus 2—Rotating Paddle) as 1.000 g of the powders were placed in 500.0 mL distilled H_2_O and stirred at 25 °C with a speed of 50 rpm. Samples of 1.0 mL were withdrawn at 5, 10, 15, 20, 30, and 45 min and diluted with ethanol to 10.0 mL in a volumetric flask. The amount of dissolved eugenol was estimated by measuring the absorbance at 282 nm with a UV spectrometer and using the calibration curve (Figure S6). It was expressed as a percentage of the eugenol content in the formulations.

### 3.11. ^1^H NMR Spectra

The ^1^H NMR spectra of pure eugenol, Soluplus^®^, and 15%E-S spray-dried formulation were recorded on a Bruker AVNEO 400 spectrometer at 400 MHz in DMSO-d6. The spectra are shown in Supplementary Materials, Figure S3.

## 4. Conclusions

The solubility of eugenol was enhanced by incorporation in the water-soluble polymers Soluplus^®^ and Lutrol F 127 using low-temperature spray drying. The morphology of the particles and the powder flowability were optimized by the addition of *myo*-inositol and Aerosil^®^ 200. Formulations containing *myo*-inositol, a biologically active compound, that could not only play the role of an excipient but also an API, and Aerosil^®^ 200 (colloidal silica) were also prepared to study the influence of these substances on the particle size and morphology, as well as the encapsulation entrapment. The highest encapsulation efficiency of 98.2% ± 1.9 is achieved for the formulations with 5% eugenol with respect to the weight of Soluplus^®^. The presence of Lutrol F 127 in the formulations leads to a more pronounced spherical shape of the particles and an improvement in the flowability of the obtained powders but notably lowers the yield to ~30–40% and the encapsulation efficiency to ~84%. The obtained DSC thermograms imply that the eugenol is embedded in the polymer matrix. FTIR spectroscopy indicates the formation of hydrogen bonds between Soluplus^®^ and eugenol. The average particle size of the obtained spray-dried formulations in water solution is in the range of 63.1 ± 2.1–82.8 ± 6.3 nm.

The formulations containing *myo*-inositol and Aerosil^®^ 200 seem to be optimal in terms of yield, powder flowability, overall amount of loaded eugenol, and encapsulation efficiency. The optimized composition is also characterized with immediate dissolution for up to 10th minute. The potential applications of the obtained formulations are as food supplements, for example, as instant powder drinks and even as adjuvant therapy, due to the antioxidant, antifungal, and antibacterial properties of eugenol.

## Figures and Tables

**Table 1:** Summary of the eugenol content in the solutions for spray drying and the estimated amount in the spray-dried powders by UV/VIS spectroscopy.

Formulation	Solids/H_2_O (g/g)	Inlet/Outlet Air Temperature (°C) ^1^	Encapsulated Eugenol (mg/g) ^2^	Theoretical Eugenol (mg/g) ^3^	Encapsulation Efficiency (%)
5% Eugenol with respect to the mass of the polymer					
5%E-S	4/40	70/30	49.5	50.4	98.2 ± 1.9
5%E-S-100	4/100	70/30	44.0	50.5	87.0 ± 1.3
5%E-S-80 °C	4/40	80/42	49.9	50.9	97.9 ± 1.8
10% Eugenol with respect to the mass of the polymer(s)					
10%E-S	4/40	70/30	93.1	100.4	92.7 ± 1.4
10%E-S-80 °C	4/40	80/41	91.1	100.2	90.9 ± 2.5
10%E-S-100	4/100	80/41	84.1	100.7	83.6 ± 2.4
10%E-S-I	8/80	80/41	90.0	100.2	89.9 ± 2.8
10%E-S-2I	12/80	80/41	89.5	100.3	89.3 ± 1.9
10%E-S-L	4/40	80/41	81.0	100.1	80.9 ± 1.5
15% Eugenol with respect to the mass of the polymer(s)					
15%E-S	4/40	70/30	136.7	150.4	90.9 ± 2.1 ^4^
15%E-S-100	4/100	70/30	124.6	150.4	82.9 ± 1.1
15%E-S-80 °C	4/40	80/41	134.0	150.6	89.0 ± 2.4
15%E-S-90 °C	4/40	90/45	133.4	150.5	88.6 ± 1.8
15%E-S-A	4.1/40	80/41	135.4	150.4	90.0 ± 0.9
15%E-S-I	8/80	80/41	134.5	150.5	89.4 ± 1.1
15%E-S-2I	12/80	80/41	134.1	150.5	89.1 ± 1.6
15%E-S-I-A	8.1/80	80/41	136.2	150.3	90.6 ± 0.8
15%E-S-2I-A	12.1/80	80/41	134.8	150.4	89.7 ± 1.5
15%E-S-L	4/40	70/30	126.7	150.8	84.1 ± 1.8
15%E-S-L-A	4.1/40	70/30	130.7	150.5	86.8 ± 1.6
15%E-S-L-I	8/80	70/30	125.8	150.3	83.7 ± 1.0
15%E-S-L-2I	12/80	70/30	125.8	150.5	84.9 ± 1.8
15%E-S-L-I-A	8.1/80	70/30	126.4	150.4	84.1 ± 1.6
15%E-S-L-2I-A	12.1/80	70/30	124.8	150.2	83.1 ± 1.5

^1^ The variation of the outlet temperature is ± 2 °C; ^2,3^ with respect to the weight of the polymer(s); ^4^ six assays were performed.

**Table 2:** Hydrodynamic diameter (D_H_) in nm and polydispersity index (PDI) of selected samples. The concentration of the measured solutions was 1 mg/mL. The standard deviation (SD) is also shown.

Formulation	D_H_ ± SD	PDI ± SD
5%E-S	66.4 ± 2.1	0.082 ± 0.075
10%E-S	68.7 ± 2.2	0.075 ± 0.082
15%E-S	64.5 ± 1.8	0.080 ± 0.057
15%E-S-A	63.1 ± 2.1	0.156 ± 0.101
15%E-S-I	69.7 ± 2.4	0.161 ± 0.107
15%E-S-2I	66.1 ± 2.8	0.202 ± 0.093
15%E-S-I-A	81.6 ± 3.9	0.163 ± 0.054
15%E-S-2I-A	74.4 ± 1.7	0.114 ± 0.064
15%E-S-L	69.2 ± 2.7	0.128 ± 0.128
15%E-S-L-A	75.2 ± 6.1	0.247 ± 0.107
15%E-S-L-I	71.0 ± 7.1	0.195 ± 0.128
15%E-S-L-I-A	82.8 ± 6.3	0.299 ± 0.084
15%E-S-L-2I	75.3 ± 1.8	0.101 ± 0.080
15%E-S-L-2I-A	77.8 ± 5.7	0.197 ± 0.165

## Data Availability

Data are available from the authors.

## Supplementary Materials

The following supporting information can be downloaded at: https://www.mdpi.com/article/10.3390/ph17091156/s1, Table S1: Loss on drying for the different formulations; Table S2: Summary of the weight of the compounds used for the preparation of the solutions for spray drying; Table S3: Design of experiments of the solutions used for spray drying containing 15% eugenol with respect to the Soluplus^®^ weight; Figure S1: Appearance of the spray-dried formulations; Figure S2: SEM micrographs of selected powders; Figure S3: ^1^H NMR spectra of pure eugenol and Soluplus^®^, and 15%E-S spray-dried formulation; Figure S4: DSC thermograms of selected powders.; Figure S5: UV spectrum of Soluplus^®^ in ethanol; Figure S6: Calibration curve used for the assay of the encapsulated eugenol
