# Supplementary material for: Efficient Improvement of Eugenol Water Solubility by Spray Drying Encapsulation in Soluplus® and Lutrol F 127
Source: Pharmaceuticals (Basel). 2024 Aug 31;17(9):1156. doi: 10.3390/ph17091156 (PMC11434763; doi:10.3390/ph17091156)
Supplement: Supplementary file 1 [file pharmaceuticals-17-01156-s001.zip › pharmaceuticals-3152952-supplementary.pdf]

Supplementary Information

Efficient improvement of eugenol water solubility by spray-drying encapsulation in Soluplus® and Lutrol F 127

Iskra Z. Koleva and Christo T. Tzachev

Faculty of Chemistry and Pharmacy, Sofia University "St. Kliment Ohridski", 1 J. Bourchier Blvd., 1164 Sofia, Bulgaria

List of content:

Table S1: Loss on drying for the different formulations.

Table S2: Summary of the weight of the compounds used for the preparation of the solutions for spray drying.

Table S3: Design of experiments of the solutions used for spray drying containing 15% eugenol with respect to the Soluplus® weight.

Figure S1: Appearance of the spray-dried formulations.

Figure S2: SEM micrographs of selected powders.

Figure S3: ^{1}H NMR spectra of pure eugenol Soluplus®, and 15%E-S spray-dried formulation.

Figure S4: DSC thermograms of selected powders.

Figure S5: UV spectrum of Soluplus® in ethanol.

Figure S6: Calibration curve used for the assay of the encapsulated eugenol.

Table S1. Loss on drying for the different formulations.

Formulation	Loss on drying (%)
5%E-S	2.56
5%E-S-100	4.00
5%E-S-80 ^{0}C	2.00
10%E-S	2.00
10%E-S-80 ^{0}C	1.93
10%E-S-100	4.00
10%E-S-I	2.98
10%E-S-2I	1.97
10%E-S-L	3.00
15%E-S	3.93
15%E-S-100	3.00
15%E-S-80 ^{0}C	4.00
15%E-S-90 ^{0}C	3.98
15%E-S-A	4.00
15%E-S-I	3.85
15%E-S-2I	3.00
15%E-S-I-A	1.97
15%E-S-2I-A	3.93
15%E-S-L	3.85
15%E-S-L-A	2.89
15%E-S-L-I	2.00
15%E-S-L-2I	4.00
15%E-S-L-I-A	3.27
15%E-S-L-2I-A	3.51

Table S2. Summary of the weight of the compounds (in g) used for the solutions for spray drying. The total amount of the polymer(s) in each solution is 4.000 g, water content (in mL), and stirring time after the addition of the polymers to the eugenol (in hours).

	Eugenol	Soluplus®	Lutrol F 127	*myo*-inositol	Aerosil® 200	H_2O	Stirring time [a]
5%E-S	0.20171	4.000	–	–	–	40	3
10%E-S	0.40146	4.000	–	–	–	40	6
10%E-S-I	0.40065	4.000	–	4.000	–	80	7
10%E-S-2I	0.40112	4.000	–	8.000	–	80	7
10%E-S-L	0.40090	3.200	0.800	–	–	40	4
15%E-S	0.60162	4.000	–	–	–	40	6
15%E-S-A	0.60141	4.000	–	–	0.100	40	7
15%E-S-I	0.60194	4.000	–	4.000	–	80	7
15%E-S-I-A	0.60135	4.000	–	4.000	0.100	80	7
15%E-S-2I	0.60189	4.000	–	8.000	–	80	7
15%E-S-2I-A	0.60157	4.000	–	8.000	0.100	80	7
15%E-S-L	0.60300	2.800	1.200	–	–	40	4
15%E-S-L-A	0.60197	2.800	1.200	–	0.100	40	5
15%E-S-L-I	0.60128	2.800	1.200	4.000	–	80	5
15%E-S-L-I-A	0.60155	2.800	1.200	4.000	0.100	80	5
15%E-S-L-2I	0.60185	2.800	1.200	8.000	–	80	5
15%E-S-L-2I-A	0.60096	2.800	1.200	8.000	0.100	80	5

[a] The stirring time depends on the amount of eugenol. When it is higher, it takes more time to incorporate in the Soluplus® micelles.

Table S3. Design of experiments of the solutions used for spray drying containing 15% eugenol with respect to the Soluplus® weight, encapsulation efficiency, EE in %, and particle size in water solution in nm of the spray-dried powders. Excipient quantities are expressed as a fraction, considering that the sum of all excipients is 1.000 g as the exact amount per each solution is shown in Table S1.

Formulation	Soluplus®	Lutrol F 127	*myo*-inositol	Aerosil® 200	EE	Particle size
15%E-S	1.000	0.000	0.000	0.000	90.9	64.5
15%E-S-A	0.976	0.000	0.000	0.024	90.0	63.1
15%E-S-I	0.500	0.000	0.500	0.000	89.4	69.7
15%E-S-I-A	0.494	0.000	0.494	0.012	89.1	66.1
15%E-S-2I	0.333	0.000	0.667	0.000	90.6	81.6
15%E-S-2I-A	0.331	0.000	0.661	0.008	89.7	74.4
15%E-S-L	0.700	0.300	0.000	0.000	84.1	69.2
15%E-S-L-A	0.683	0.293	0.000	0.024	86.8	75.2
15%E-S-L-I	0.350	0.150	0.500	0.000	83.7	71.0
15%E-S-L-I-A	0.346	0.148	0.494	0.012	84.1	82.8
15%E-S-L-2I	0.233	0.100	0.667	0.000	84.9	75.3
15%E-S-L-2I-A	0.231	0.099	0.661	0.008	83.1	77.8

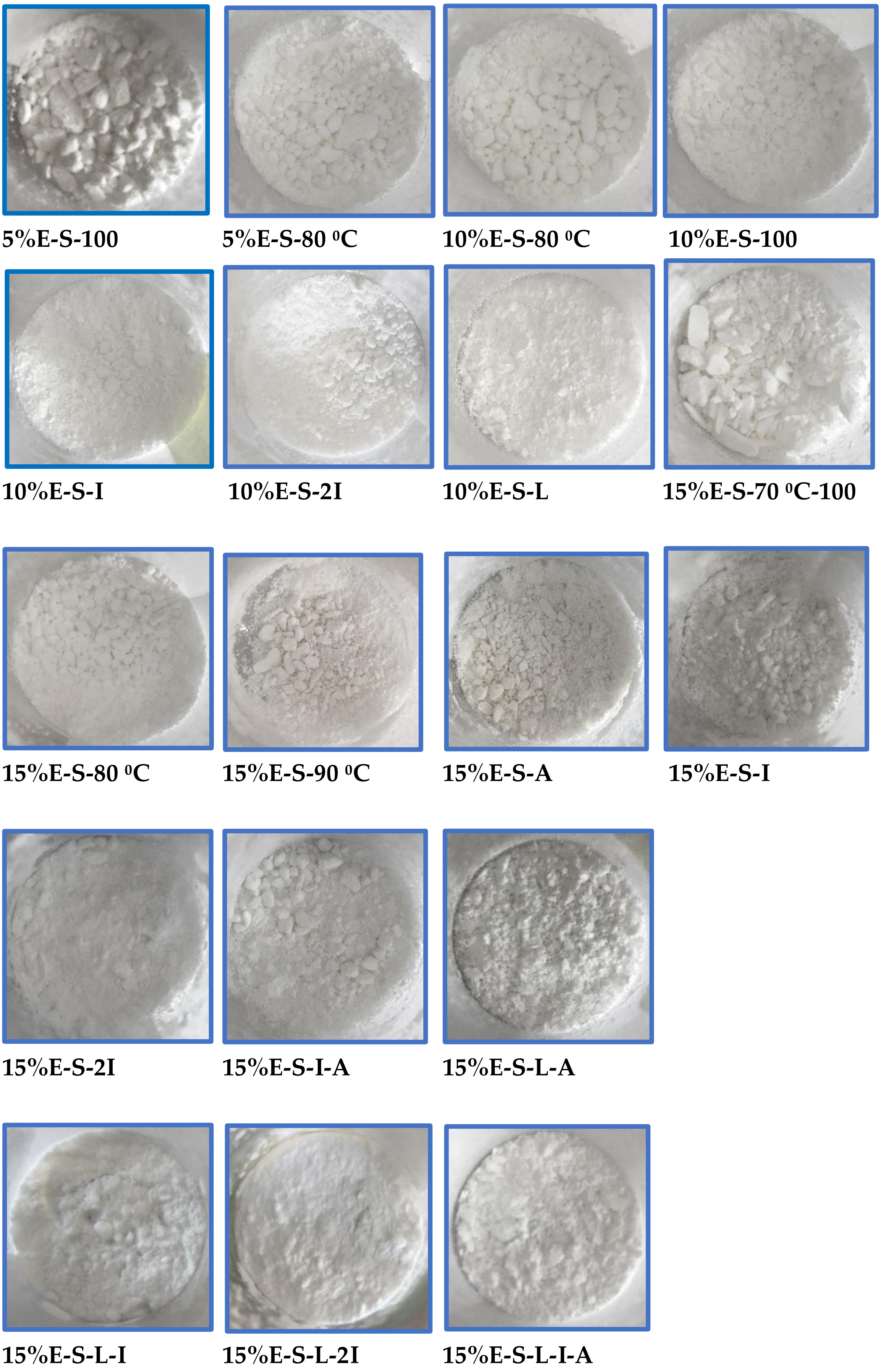

Figure S1. Appearance of the spray-dried formulations.

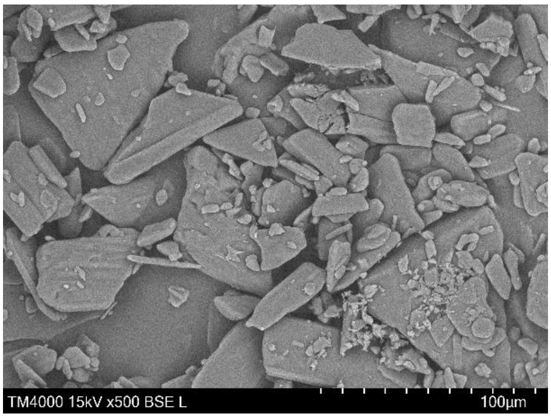

Inositol

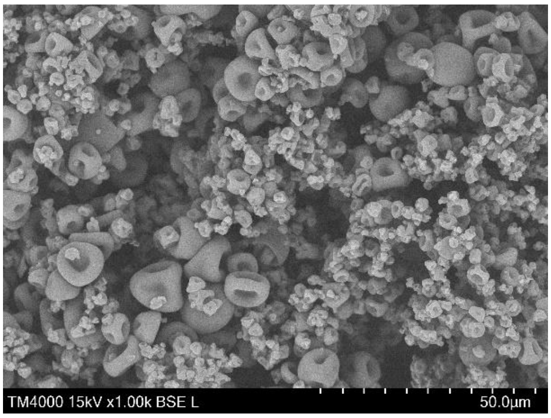

15%E-S-70^{0}C-100

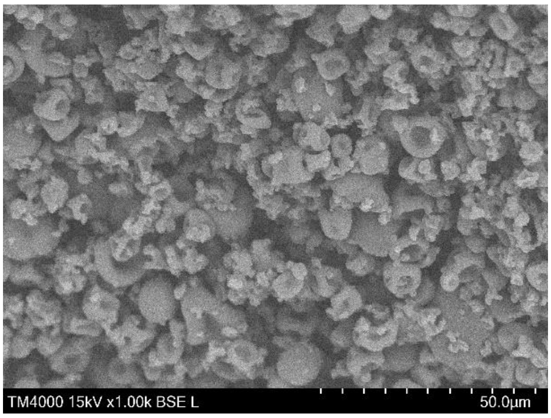

15%E-S-80^{0}C

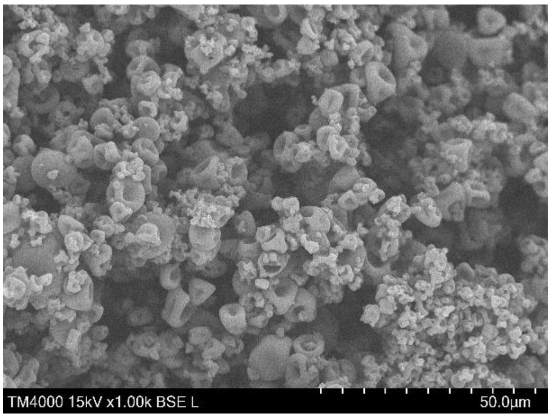

15%E-S-90^{0}C

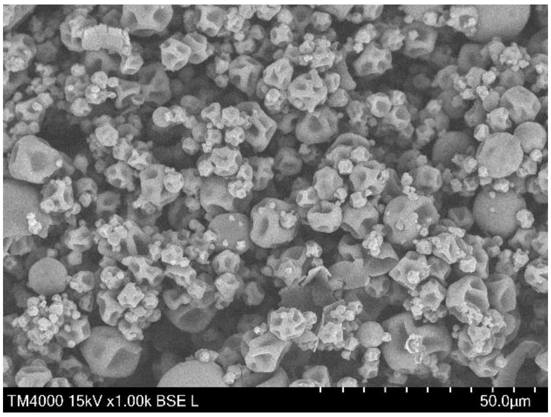

15%E-S-I

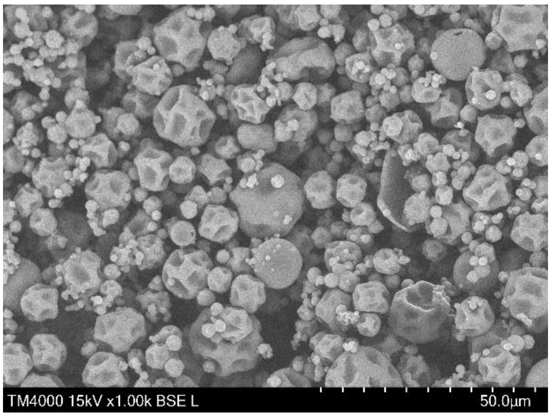

15%E-S-2I

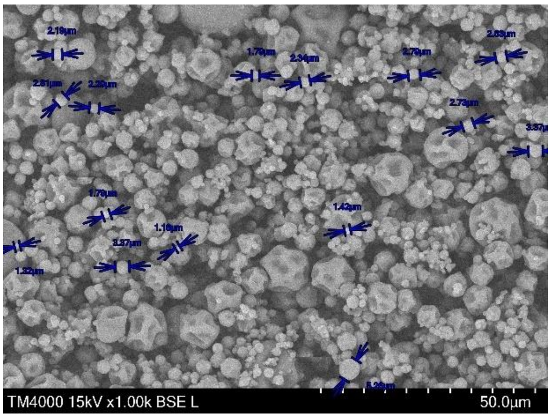

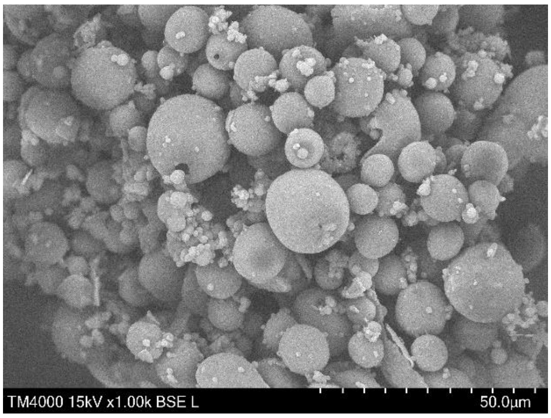

15%E-S-2I

15%E-S-L-I

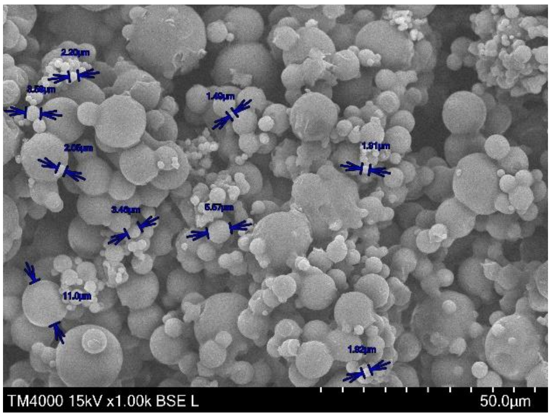

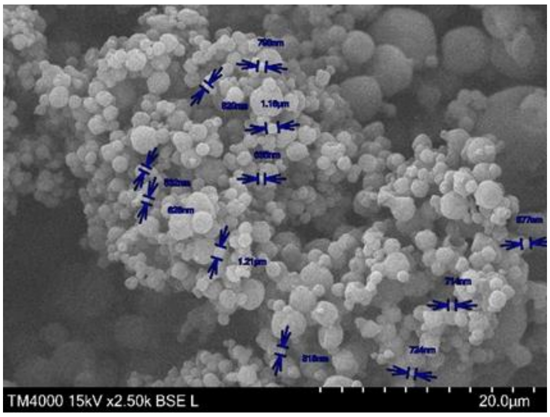

15%E-S-L

Figure S2. SEM micrographs of selected powders.

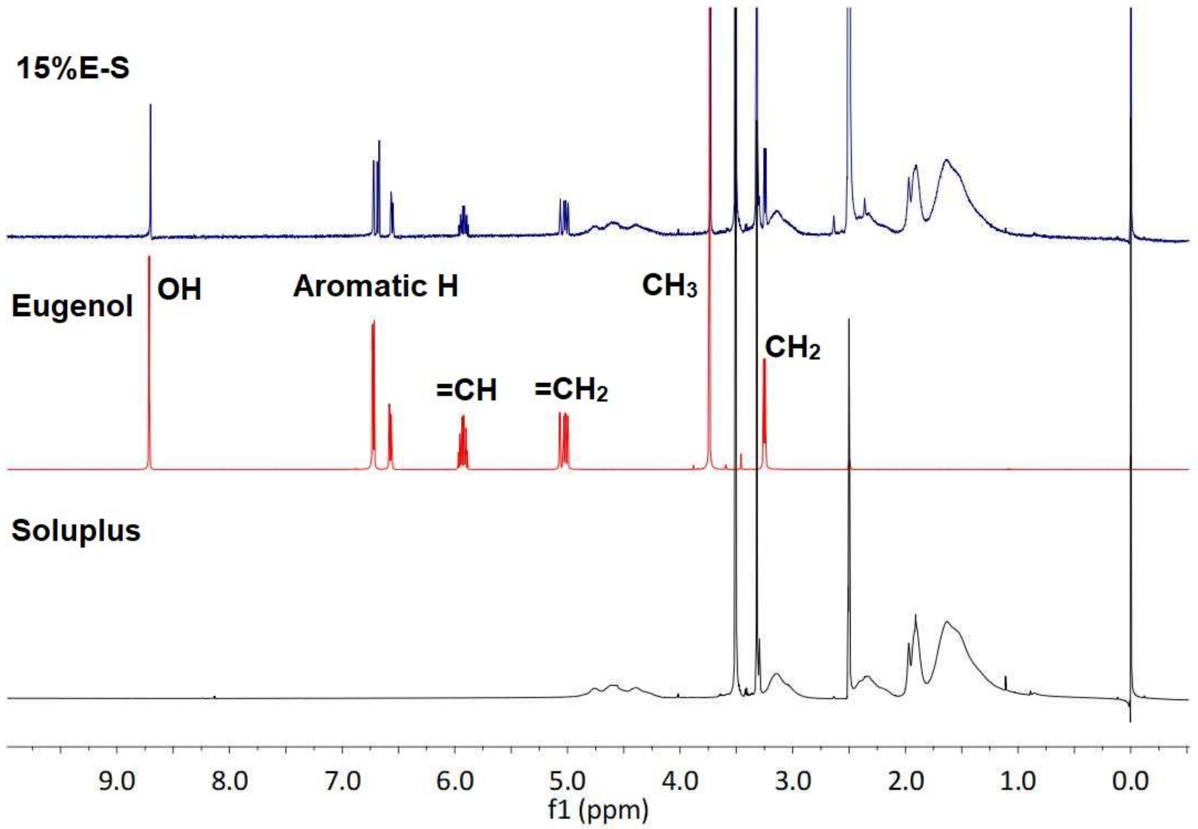

Figure S3. ^1H NMR spectra in DMSO-d6 of pure eugenol and Soluplus®, and 15%E-S spray-dried formulation. The signals of the eugenol protons are denoted on its spectrum.

^1H NMR eugenol: δ = 3.25 ppm (d, 2H); δ = 3.74 ppm (s, 3H), δ = 5.03 ppm (dd, 2H), δ = 5.92 ppm (m, 1H); aromatic protons – δ = 6.57 (dd, 1H) and 6.71 ppm (t, 2H); δ = 8.71 ppm (s, 1H).

^1H NMR Soluplus®: δ = 1.63 ppm (CH_2), δ = 1.90 ppm (CH_3 next to the ester carbonyl group), 2.34 ppm (CH_2 next to the amide carbonyl group); δ = 3.14 ppm (CH_2 next to the N atom), δ = 3.51 ppm (CH_2 from PEG); δ = 3.32 ppm (H_2O).

^1H NMR 15%E-S: The signals listed above for eugenol and Soluplus® are observed.

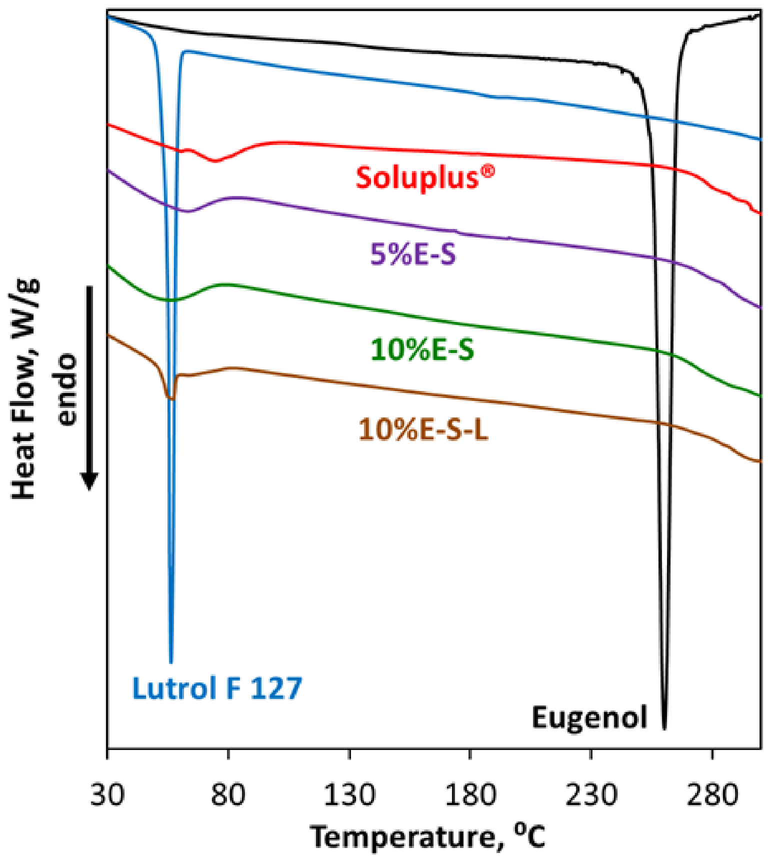

Figure S4. DSC thermograms of selected powders.

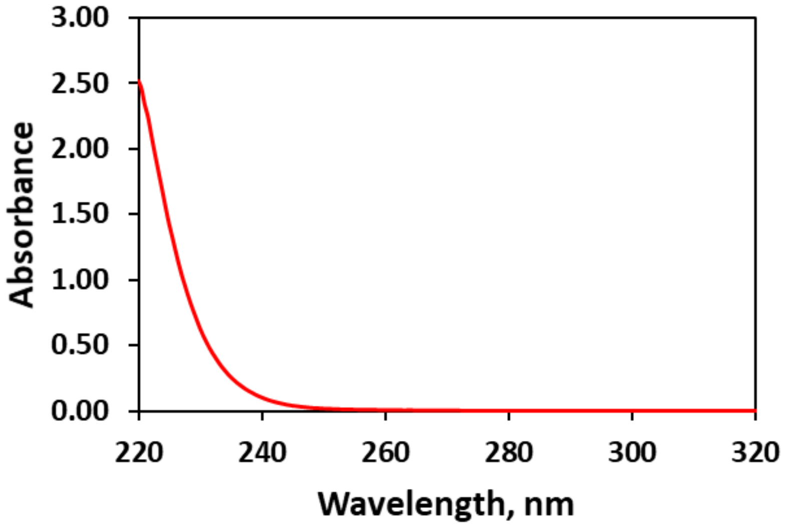

Figure S5. UV spectrum of Soluplus® (0.2 mg/mL) in ethanol. The absorbance at 282 nm is 0.000.

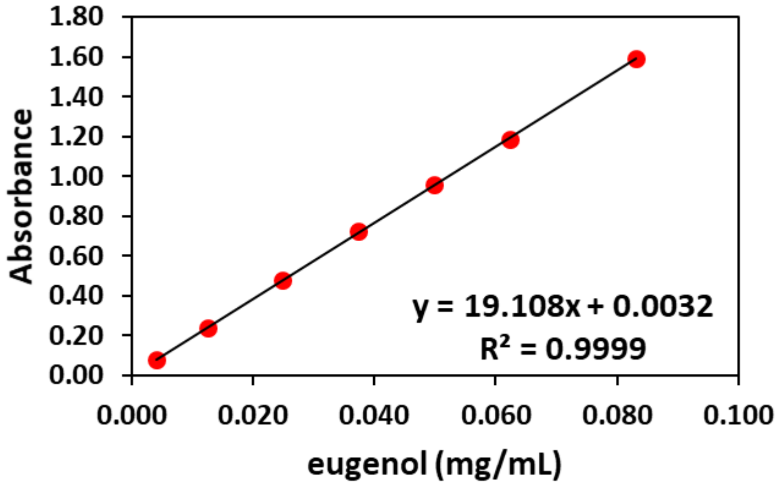

Figure S6. Calibration curve used for the assay of the encapsulated eugenol.
